# Supplementary material for: Comprehensive Identification and Functional Analysis of Stress-Associated Protein (SAP) Genes in Osmotic Stress in Maize
Source: Int J Mol Sci. 2022 Nov 13;23(22):14010. doi: 10.3390/ijms232214010 (PMC9692755; doi:10.3390/ijms232214010)
Supplement: Supplementary file 1 [file ijms-23-14010-s001.zip › ijms-2002611-supplementary.pdf]

Figure S1. Multiple alignments of amino acid sequences of ZmSAPs

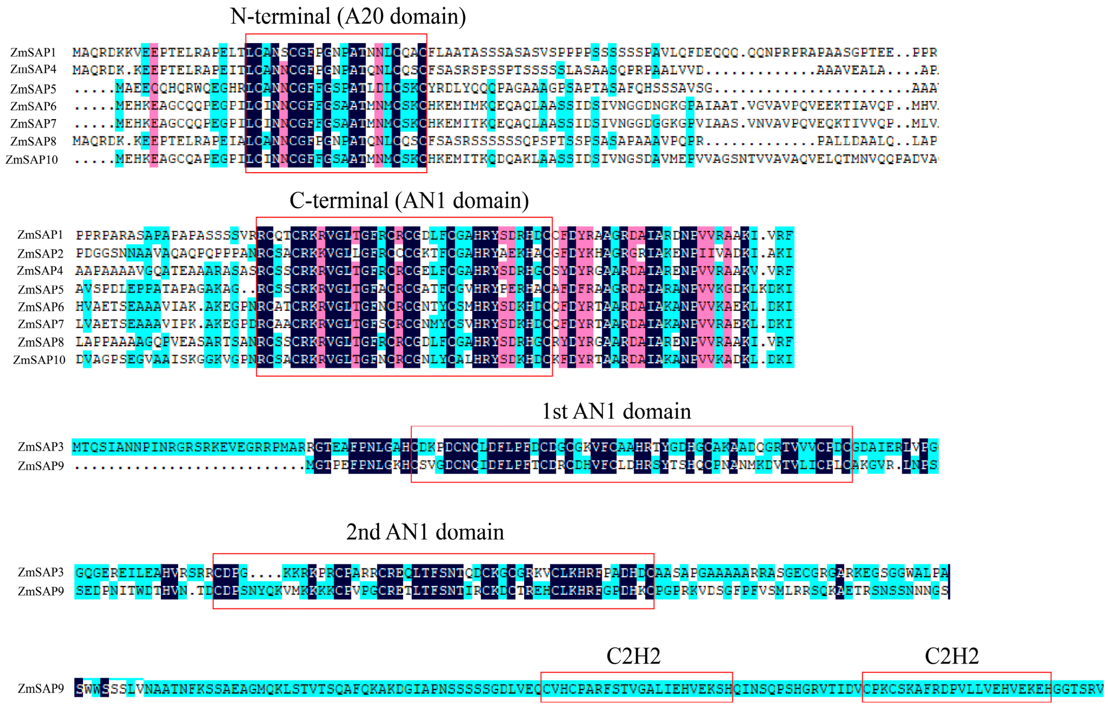

Figure S2. Phylogenetic evolutionary tree of ZmSAPs.

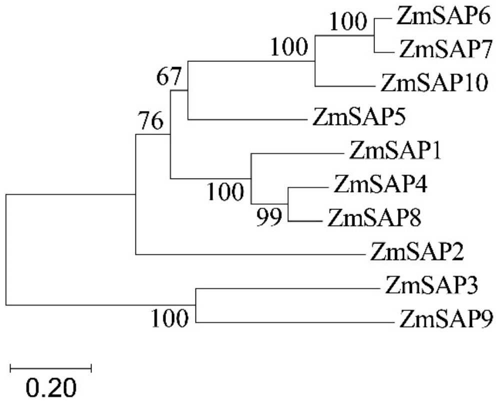

Figure S3. Expression profiles of *ZmSAPs* in different development stages of maize.

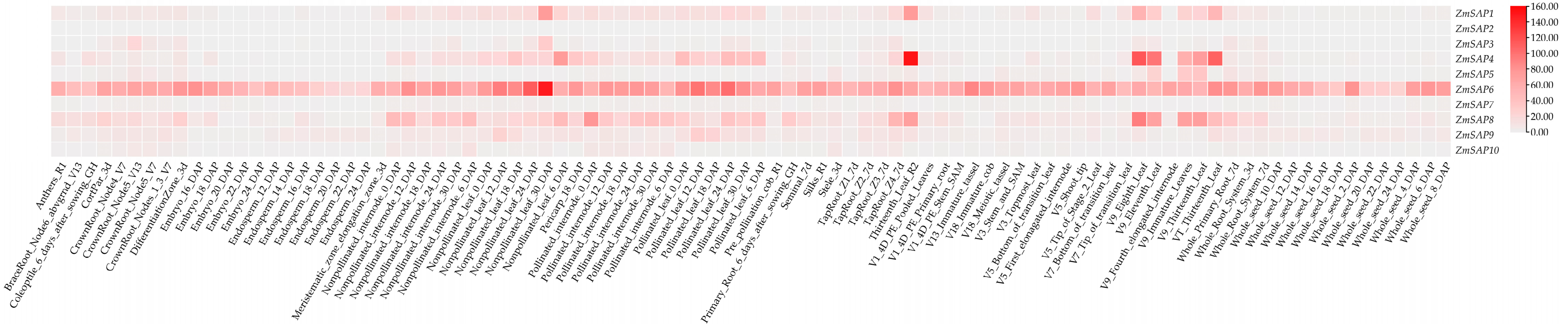

Figure S4. Phenotype of yeast under drought stress mimicking by mannitol.

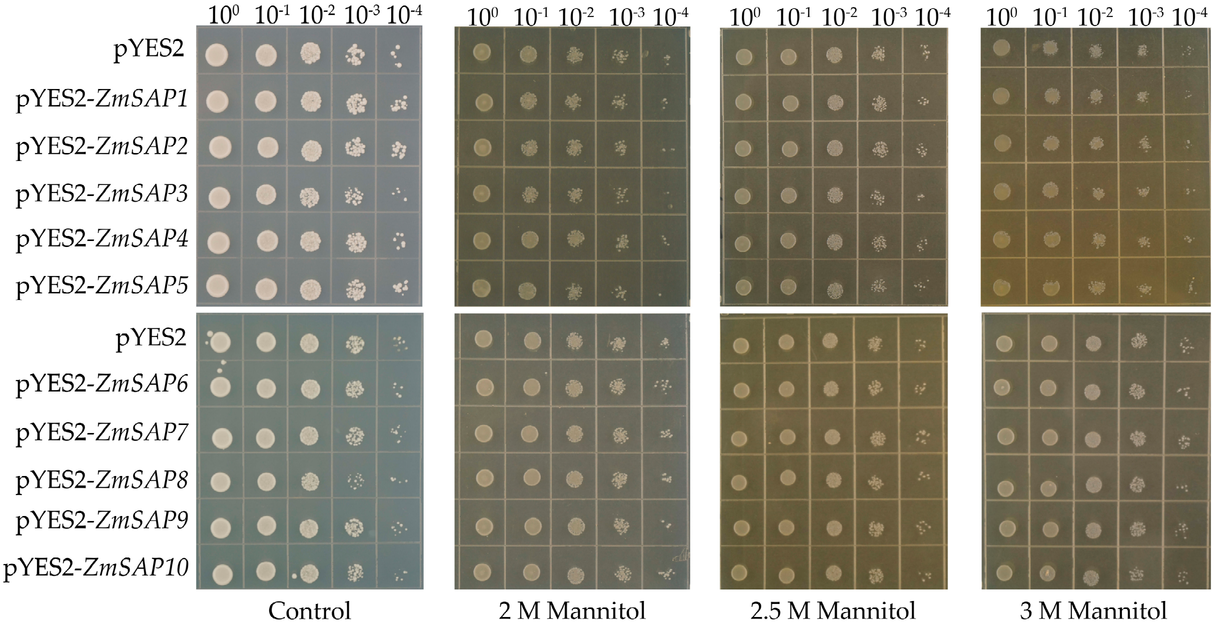

Table S1. Collinearity relationship between SAP genes in maize and rice

Name of *ZmSAP*	Name of *OsSAP*
ZmSAP1	*OsSAP1*
ZmSAP 1	*OsSAP5*
ZmSAP 2	*OsSAP14*
ZmSAP 3	*OsSAP17*
ZmSAP 4	*OsSAP1*
ZmSAP 4	*OsSAP5*
ZmSAP 5	*OsSAP8*
ZmSAP 6	*OsSAP8*
ZmSAP 6	*OsSAP4*
ZmSAP 7	*OsSAP8*
ZmSAP 7	*OsSAP4*
ZmSAP 9	*OsSAP16*
ZmSAP 10	*OsSAP8*
ZmSAP 10	*OsSAP4*

Table S2. Primers used in this study.

Gene	Primer sequence (5-3)	Assay
ZmSAP1	ACGACTGCTGCTTCGACTAC<br>ATCCGAGACGCCTAGAACCT	for RT-qPCR
	CTTGGTACCGAGCTCGGATCCATGGCTCAGCGCGACAAG<br>CCCTCTAGATGCATGCTCGAGGAACCTAACGATCTTGGCGGC	for pYES2-*ZmSAP1* construction
ZmSAP 2	CTGTTTTCGAGACAACGCCG<br>TTATTGCTGCCTCCGTCAGG	for RT-qPCR
	CTTGGTACCGAGCTCGGATCCATGACGCAGAAGCGGAAGAG<br>CCCTCTAGATGCATGCTCGAGAATTTTGGCGATCTTGTCGG	for pYES2-*ZmSAP2* construction
ZmSAP 3	GAAAAGAGGTAGAGGGGCGG<br>AAGTCGAGCTGGTTGCAGTC	for RT-qPCR
	CTTGGTACCGAGCTCGGATCCATGACCCAATCAATCGCTAACAA<br>CCCTCTAGATGCATGCTCGAGGAAGATCTTGAGGCTCCGGATG	for pYES2-*ZmSAP3* construction
ZmSAP 4	CGGACAAGGTCTTCGGTGTT<br>CCACTGCACCACCACCATTA	for RT-qPCR
	CTTGGTACCGAGCTCGGATCCATGGCGCAGCGCGACAAG<br>CCCTCTAGATGCATGCTCGAGGAACCTAACGACCTTGGCCG	for pYES2-*ZmSAP4* construction
ZmSAP 5	ACGGATCGTCGGATTGTACG<br>TCAGTAAACGGCCAGCGATT	for RT-qPCR
	CTTGGTACCGAGCTCGGATCCATGGCGGAGGAGCAGCAG<br>CCCTCTAGATGCATGCTCGAGGATCTTGTCCTTGAGCTTATCGC	for pYES2-*ZmSAP5* construction
ZmSAP 6	CCCCAGTCGAGTCACGAAAA<br>ACGATACCGACCCAAACCAC	RT-qPCR
	CTTGGTACCGAGCTCGGATCCATGGAACACAAGGAGGCCG<br>CCCTCTAGATGCATGCTCGAGGATCTTGTCAAGCTTCTCCGCC	for pYES2-*ZmSAP6* construction
ZmSAP 7	GTGTTTTCAGGCCACTGCTC<br>GCAGAGGTTGCTTCCTCCTA	for RT-qPCR
	CTTGGTACCGAGCTCGGATCCATGGAACACAAGGAGGCGG<br>CCCTCTAGATGCATGCTCGAGGATCTTGTCGAGCTTCTCCGC	for pYES2-*ZmSAP7* construction
ZmSAP 8	TAGGTTCTGATTCGACCGGC<br>GATTTCGCATGCCTTTCCCC	for RT-qPCR
	CTTGGTACCGAGCTCGGATCCATGGCGCAGCGCGACAAG<br>CCCTCTAGATGCATGCTCGAGGAACCTAACGATCTTGGCCGC	for pYES2-*ZmSAP8* construction
ZmSAP 9	TCACGGTCTCACTCTCACCT<br>AAACGGACGGGACACGAAAT	for RT-qPCR
	CTTGGTACCGAGCTCGGATCCATGGGCACGCCGGAGTTC<br>CCCTCTAGATGCATGCTCGAGCACTCTTGACGTTCCTCCATGC	for pYES2-*ZmSAP9* construction
ZmSAP 10	CTTTATCTCTCTCCCCACGCC<br>CGATTCCTCTCCGACGATCC	for RT-qPCR
	CTTGGTACCGAGCTCGGATCCATGGAGCACAAGGAGGCTGG<br>CCCTCTAGATGCATGCTCGAGGATCTTGTCGAGCTTGTCTGCC	for pYES2-*ZmSAP10* construction
